# Glabridin attenuates lipopolysaccharide-induced acute lung injury by inhibiting p38MAPK/ERK signaling pathway

**DOI:** 10.18632/oncotarget.14277

**Published:** 2016-12-27

**Authors:** Li-peng Zhang, Yan Zhao, Guo-juan Liu, Da-gang Yang, Yi-huan Dong, Li-hua Zhou

**Affiliations:** ^1^ Department of Critical Care Medicine, The Affiliated Hospital of Innner Mongolia Medical University, Hohhot 010050, China; ^2^ Department of Neurology, The people’ Hospital of Inner Mongolia, Hohhot 010050 China

**Keywords:** glabridin, acute respiratory distress syndrome, p38MAPK, ERK

## Abstract

**Background:**

Acute respiratory distress syndrome (ARDS) is a complication caused by pulmonary and/or external factors. In this study, we investigated the protective mechanisms of glabridin in lipopolysaccharide (LPS) induced ARDS in rats.

**Results:**

GLA treatment at dose of 30 mg/kg decreased LPS-induced lung W/D ratio and alleviated evident lung histopathological changes. Expressions of TNF-α and IL-18 were suppressed by GLA in plasma. The levels of SPA, MDA and NO in lung were down-regulated significantly in groups administrated with GLA. While the SOD level increased after GLA administration. Additionally, the attenuation of inflammatory responses by GLA was closely associated with p38MAPK/ERK pathway, and the expressions of protein p-p38MAPK and pERK were inhibited by GLA in LPS-induced ARDS rats.

**Materials and Methods:**

Sixty-four Wistar rats were randomly assigned into control group, Glabridin (GLA) alone group, LPS groups (6 h, 12 h, 24 h), GLA with LPS groups (6 h, 12 h, 24 h). ARDS was induced in rats by intraperitoneal administration of LPS (10 mg/kg). The degree of lung edema was evaluated by calculating the wet/dry weight ratio. The levels of inflammatory mediators, tumor necrosis factor-α (TNF-α) and interleukin-18 (IL-18) were assayed by enzyme-linked immunosorbent assay (ELISA). Surfactant protein A (SPA), malondialdehyde (MDA), nitric oxide (NO) and superoxide dismutase (SOD) were analyzed. Pathological changes of lung tissues were observed by H&E staining. The protein expression of p38MAPK and ERK was detected using immunohistochemical techniques. Lung phosphorylated p38MAPK (p-p38MAPK) and pERK protein expression changes were detected by Western blotting.

**Conclusions:**

Glabridin significantly ameliorated the lung injury induced by LPS in rats via the inhibition of p38MAPK and ERK signaling pathway, antioxidant effect and reducing inflammation.

## INTRODUCTION

Sepsis is a common complication among clinical critical patients, which may lead to multiple organ dysfunction syndrome (MODS). The lung is the most common target organ of early injury. Acute respiratory distress syndrome (ARDS) is a complication that arises in the intensive care unit and contribute to remarkable morbidity and mortality. It is characterized by increased pulmonary alveolar capillary membrane permeability caused by various pathogenic factors. Due to the rapid progression of the disease and lack of effective treatment, it is urgent to develop and validate pharmaceutical drugs for the treatment of ARDS [1].

Traditional Chinese herbal medicine glabridin (GLA) is a kind of flavonoid extracted from licorice flavonoids. Glabridin has been thought to be have benefits ranging from anti-inflammatory, antioxidant and estrogen-like effect to inhibition of tyrosine activity [2]. The lipopolysaccharide (LPS) components of Gram-negative bacteria are reported to be an important risk factor for ARDS. Mitogen activated protein kinases (MAPKs) signaling pathway is the major signal transduction pathways regulating the production of inflammatory cells and has a close relationship with the LPS-induced ARDS [3]. Therefore, the present study aims to investigate the protective effect and possible mechanism of glabridin on ARDS through LPS-induced ARDS model in rats.

## RESULTS

### Effects of glabridin on lung W/D ratio in LPS-induced ALI rats

The lung W/D ratio was determined to evaluate the pulmonary edema. Administration of LPS increased the level of lung W/D ratio (*P* < 0.05), most obviously at 12 h. Notably, glabridin significantly reduced the pulmonary edema (*P* < 0.05), but the effect is less than 24 h (Table 1).

**Table 1:** Lung W/D ratio and SPA, IL-6 and TNF-α levels in plasma (n = 8)

Group	W/D	plasma		
		SPA(ng/L)	TNF-a (μg/L)	IL-18 (μg/L)
Control Group	3.69 ± 1.12	16.01 ± 1.33	38.02 ± 8.35	32.22 ± 3.71
GLA	3.82 ± 2.01	16.41 ± 2.01	36.76 ± 6.72	31.42 ± 3.18
LPS				
6 h	5.55 ± 0.73^a^	18.13 ± 2.53	106.26 ± 30.37^a^	49.21 ± 6.35^a^
12 h	5.82 ± 0.94^a^	22.3 ± 2.79^a^	105.91 ± 28.29^a^	49.37 ± 13.27^a^
24 h	5.63 ± 0.47^a^	20.63 ± 3.67 ^a^	93.04 ± 21.66^a^	42.34 ± 14.01^a^
LPS+GLA				
6 h	4.31 ± 1.79^b^	16.81 ± 3.34	58.24 ± 19.86^b^	36.35 ± 27.94^b^
12 h	4.30 ± 0.67^b a^	18.82 ± 1.88^b^	50.67 ± 9.42^b^	36.82 ± 12.56^b^
24 h	4.25 ± 0.96^a^	18.43 ± 1.66^b^	61.50 ± 21.82^b^	39.36 ± 8.29^b^

The table indicates the change of W/D, SPA, IL-18, TNF-a among the groups, compared with control group ^a^*P* < 0.05, and compare with LPS group ^b^*P* < 0.05.

### Glabridin suppresses inflammatory cytokines in LPS-induced ALI rats

To investigate the anti-inflammatory effects of glabridin, the concentrations of inflammatory cytokines SPA, TNF-α and IL-18 were identified. The levels of TNF-α and IL-18 were significantly elevated after LPS administration (*P* < 0.05), most obviously at 6 h. Glabridin significantly inhibited LPS-induced inflammatory cytokine productions (*P* < 0.05), and the efficacy was stronger in less than 12 h (Table 1). Therefore, glabridin could suppress inflammatory mediators in LPS-induced ALI rats.

### Effects of glabridin on MDA, NO and SOD in LPS-induced ALI rats

The levels of MDA, NO significantly increased in LPS 12 h and 24 h groups (*P* < 0.05), while the level of SOD in LPS alone groups decreased (*P* < 0.05). The levels of MDA decreased, and the levels of SOD significantly increased in LPS+GLA groups compared with LPS groups. (Table 2)

**Table 2:** SOD, MDA and NO levels in lung tissues (n = 8)

Group	Lung tissue		
	SOD(U/mgprot)	MDA(nmol/mgprot)	NO(umol/L)
Control Group	126.15 ± 11.36	1.67 ± 0.24	17.82 ± 1.59
GLA	125.28 ± 12.46	1.59 ± 0.36	16.98 ± 1.93
LPS			
6 h	68.04 ± 5.57^a^	3.19 ± 0.17 ^a^	19.81 ± 2.76
12 h	76.21 ± 12.71^a^	3.75 ± 0.39 ^a^	27.61 ± 2.37^a^
24 h	77.45 ± 15.59^a^	4.45 ± 1.19 ^a^	22.14 ± 3.37^a^
LPS+GLA			
6 h	95.39 ± 7.73^b^	2.47 ± 0.26^b^	19.20 ± 2.78
12 h	95.39 ± 7.73^b^	2.91 ± 0.57^b^	20.82 ± 0.72
24 2h	95.39 ± 7.73^b^	3.59 ± 1.11^b^	21.19 ± 1.02

The table indicates the change of SOD, MDA, NO among the groups, compared with control group ^a^*P* < 0.05, and compare with LPS group ^b^*P* < 0.05.

### Effects of glabridin on LPS-induced lung histopathologic changes

**Figure 1:**
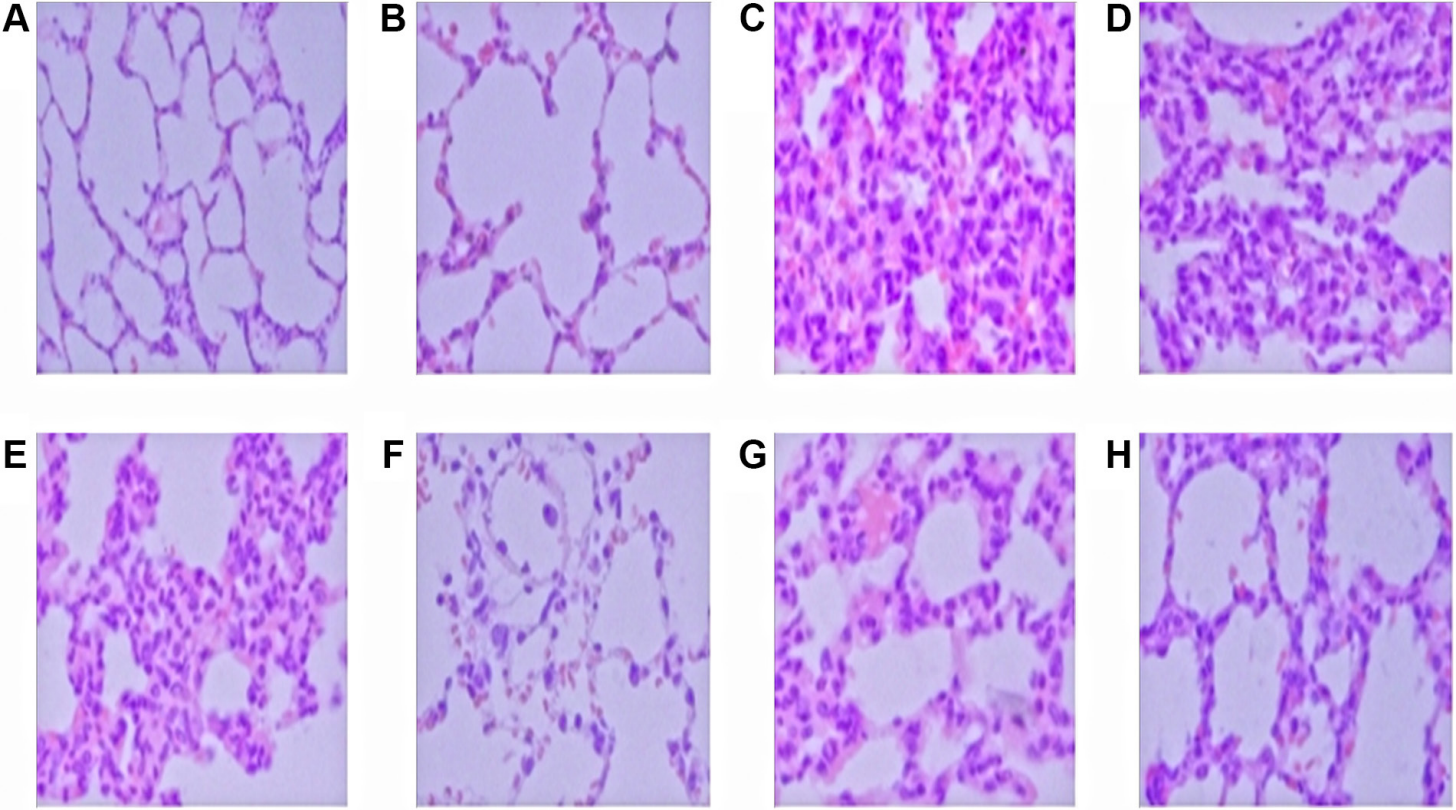
Control group (A) and GLA group (B) The structure was intact, with clear alveolar space, no congestion in alveolar wall. 6 h, 12 h, 24 h LPS group (**C**, **D**, **E**) show obvious inflammatory cell infiltration, alveolar hemorrhage and structural damage. 6 h, 12 h, 24 h LPS+GLA group (**F**, **G**, **H**) structural damage were significantly reduced. ( original magnification ×400).

Under light microscopy, the normal H&E stained lung tissue was intact, with clear alveolar space and no congestion in alveolar wall (Figure 1A). Diffuse thickening of the alveolar wall, inflammatory cells infiltration, partial alveolar hemorrhage and structural damage could be observed in all LPS alone groups (Figure 1C–1E). The most typical changes were detected at 12 h after LPS administration (Figure 1C). Administration of glabridin effectively alleviated the destruction of lung structure (Figure 1F–1H). Thus, glabridin can ameliorate the lung injury induced by LPS in rats.

### Phosphorylated p38MAPK/ERK expression in LPS-induced rats

**Figure 2:**
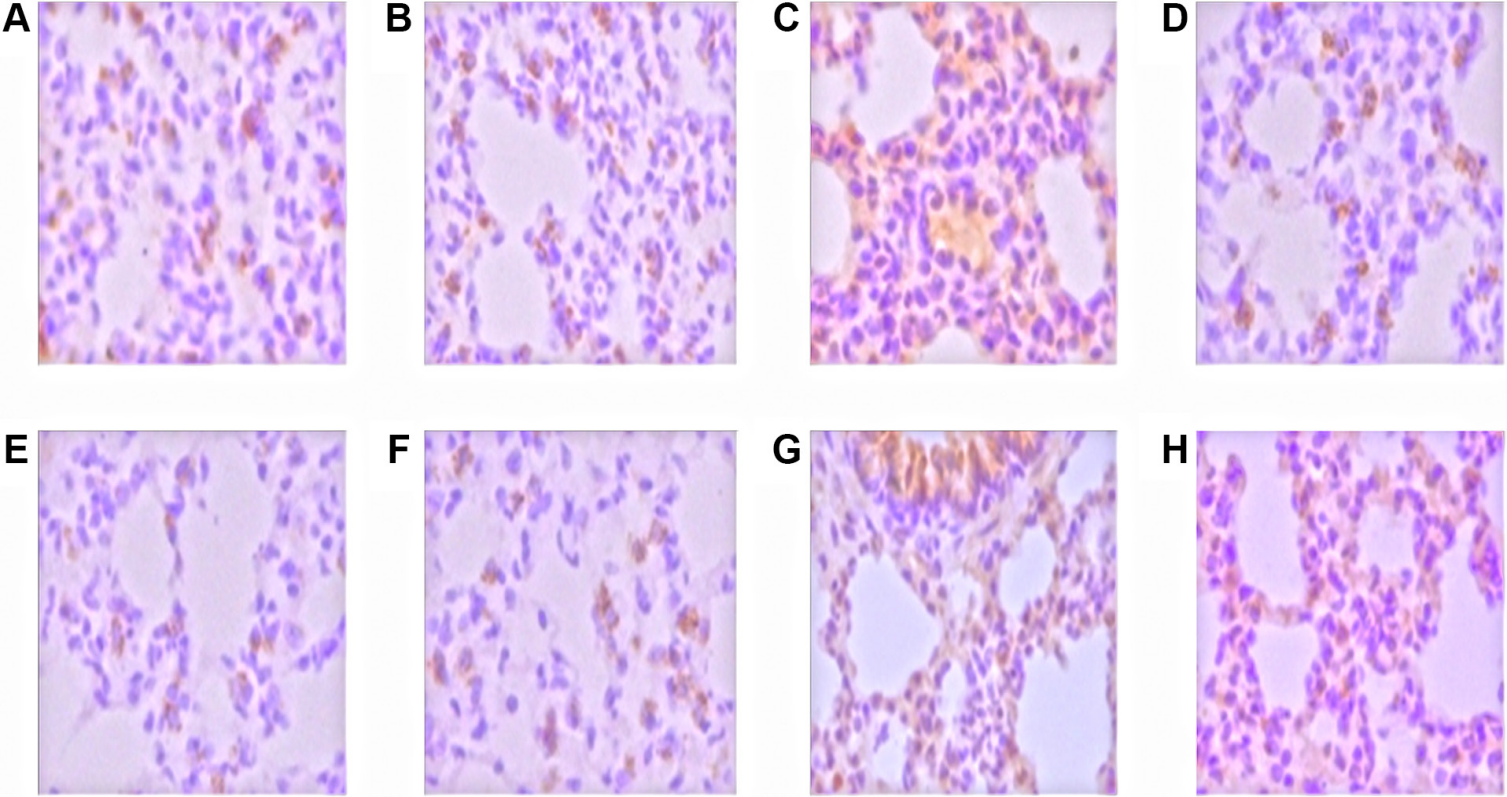
The expression of p38MAPK and ERK in the nucleus was stained with brown and yellow Control group (**A**) and GLA group (**B**), the p38MAPK expression was weak and scattered in the airway epithelium and alveolar epithelial cells; LPS group (**C**), p38MAPK expressed in inflammatory cells, alveolar epithelial cells, airway epithelial cells and vascular endothelial cells; LPS+GLA group (**D**), p38MAPK expressed cells decreased significantly. Control group (**E**) and GLA group (**F**), ERK expression was weak, distributed in the cytoplasm of airway epithelial cells and alveolar epithelial cells; LPS group (**G**), ERK expressed in inflammatory cells, alveolar epithelial cells, airway epithelial cells and vascular endothelial cells; LPS+GLA group (**H**), ERK expressed cells decreased significantly. (original magnification ×400)

Positive signal of p38MAPK/ERK was weak and occasionally could be found in the airway epithelium and alveolar epithelial cells (Figure 2A, 2B, 2E, 2F). The expression of phosphorylated p38MAPK/ERK positive signal in LPS groups was significantly enhanced (Figure 2C, 2G), which mainly distributed in airway epithelium cells, infiltration of inflammatory cells and vascular endothelial cells. Both the cytoplasm and cell nucleus were positive. The distribution of p38MAPK/ERK positive cells in LPS+GLA groups was similar to that in LPS alone groups (Figure 2D, 2H). However, the positive expression cells were significantly decreased and the signal was lower than that in LPS+GLA groups.

### Glabridin inhibited p-p38MAPK/pERK expression in LPS-induced rats

**Figure 3:**
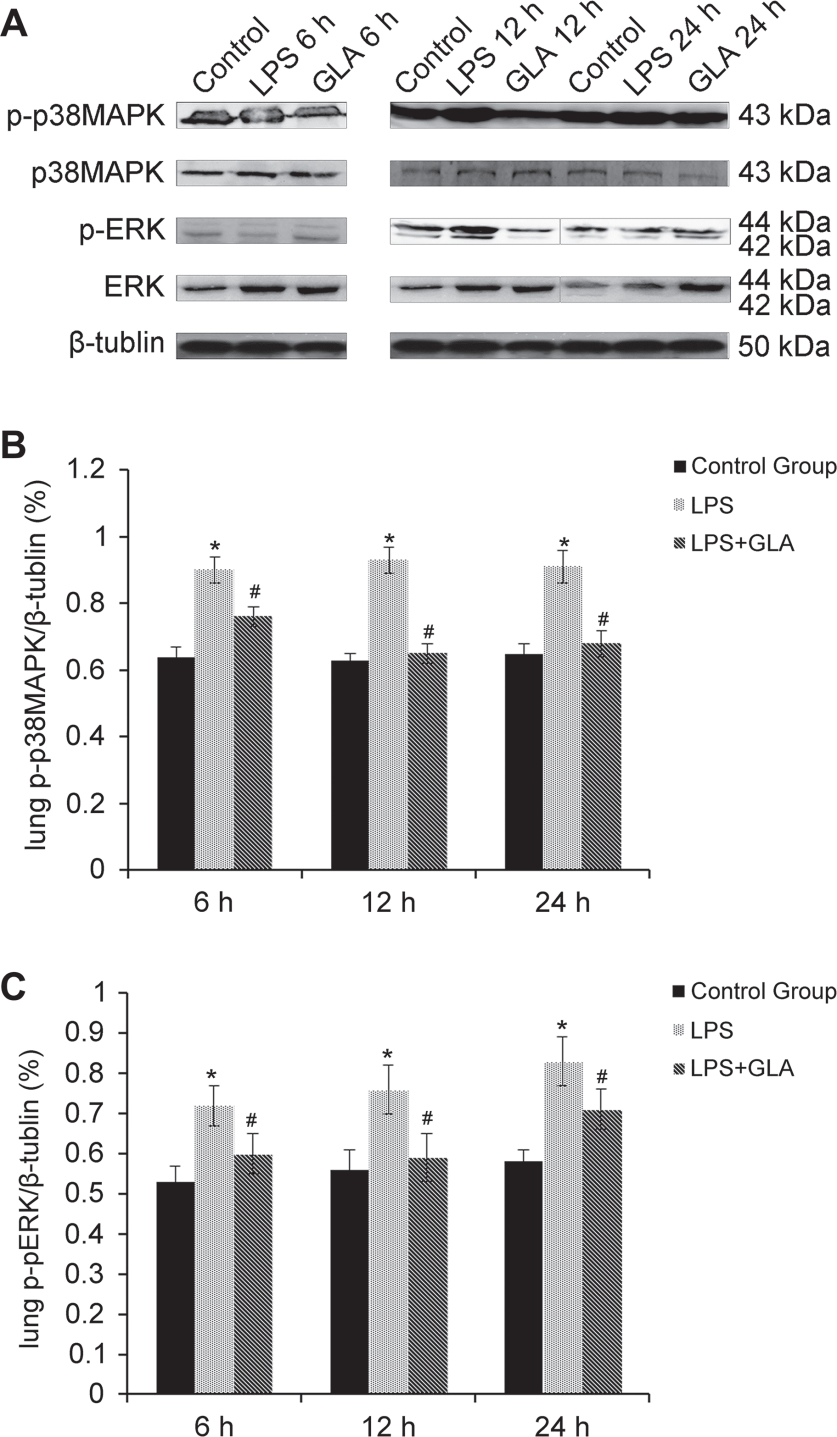
The expression of p-p38MAPK in LPS group significantly increased, p-p38MAPK induced by LPS was inhibited by glabridin The expression of p38MAPK was not significantly different among these groups. The expression of both ERK1/2 and pERK1/2 in LPS group increased, and ERK1/2 and Perk1/2 induced by LPS was significantly inhibited by glabridin.

Western blot analysis was used to determine the expression of p-p38MAPK in lung tissues. As illustrated in Figure 3, rats challenged with LPS showed significant increase of the expression of p-p38MAPK. However, glabridin markedly inhibited the expression of p-p38MAPK in LPS-induced rats. The expression of p38MAPK was not significantly different among these groups. The expression of both ERK1/2 and pERK1/2 in LPS alone groups increased. ERK1/2 and pERK1/2 induced by LPS were significantly inhibited by glabridin.

## DISCUSSION

Acute respiratory distress syndrome is caused by pulmonary injury and / or extrapulmonary injury. It is the earliest and most common tissue injury of sepsis. At present, the release of excessive inflammatory mediators and the activation and aggregation of the inflammatory cells are considered to be the key pathogenesis of sepsis and ARDS [1]. Current treatment is limited to mechanical ventilation and avoiding fluid overload. Only several drugs are proven to be effective in the treatment of lung injury. Glabridin, a traditional Chinese herbal, is the main ingredient of licorice flavonoids with broad biological activity: antioxidant [4], anti-inflammatory [5], regulation of energy metabolism, antibacterial [6] and skin whitening, etc. In murine macrophages and microglia, licorice flavonoids have been shown to inhibit the release of prostaglandin E2 (PGE2), IL-1, nitric oxide (NO) and TNF-α. Therefore, Glabridin may show benefits in the treatments of ARDS. This study mainly investigates the role of glabridin in the pathogenesis of ARDS induced by LPS in rats and its possible mechanism.

It has been proven *in vivo* that glabridin can protect hygromycin-resistant BDF1 transgenic mice against LPS-induced sepsis by reducing the production of various inflammatory mediators such as TNF-α and NO [7] . Glabridin can inhibit the infiltration of inflammatory cell and the release of inflammatory mediators. Thus, oxidative damage to neutrophils and LPS-induced inflammatory response can be effectively reduced [8, 9]. This study shows that glabridin can attenuate the lung injury induced by LPS in rats. Compared with the LPS alone groups, the pathological changes of lung tissue and the ratio of lung W/D of LPS+GLA groups were reduced, and the concentrations of TNF-α, IL-18 and NO significantly decreased. These results are consistent with previous studies. Glabridin can inhibit the inflammatory mediators to reduce the inflammatory response and alleviate LPS-induced lung injury.

Yang's [10] study have shown that the anti-inflammatory mechanism of flavonoids in licorice mainly inhibit ERK phosphorylation levels in ERK/MAPK pathway. It can down-regulate the expression of iNOS and COX-2 gene and protein, but independent of JNK and P38 pathway. The results of immunohistochemistry and Western-bolt analysis in this study showed that glabridin could reduce the phosphorylation of p38MAPK and ERK in lung tissue induced by LPS. ERK and p38MAPK signaling pathways are well known for their relationship with inflammatory response and lung injury. Therefore, our study confirmed that p38MAPK and ERK signaling pathway could be inhibited by glabridin. It is different from Yang's research that glabridin can inhibit the expression of ERK signaling pathway only.

Peroxidation is also one of the causes of acute lung injury. The concentrations of MDA and NO can be used to predict the level of peroxidation. SOD has antioxidant effect by scavenging the oxygen free radicals *in vivo*. Yu et al. [11] found that glabridin can significantly reduce the brain MDA concentration and enhance the activity of endogenous antioxidant SOD. Yokota et al. [12] found that glabridin can inhibit the superoxide anion production between the concentration of 0.333 ug/ml and 33.3 ug/ml. 75.7% of superoxide anion production was inhibited under the concentration of 33.3 ug/ml. Yehuda's study confirmed that glabridin had potential to enhance antioxidant defense [13].

Administrated with glabridin, the content of MDA in the lung homogenate decreased significantly and the content of SOD increased obviously. The pathological changes of the lung tissue in the LPS+GLA groups were less severe than that in the LPS alone groups, suggesting that glabridin yields anti-oxidation activity both in brain and lung tissue. This study confirmed that glabridin could alleviate LPS-induced lung injury in rats through its strong antioxidant effects, which is consistent with previous studies [6]. NO is more than an inflammatory mediator. Studies have shown that LPS can stimulate the rat lung tissue iNOS expression and the excessive release of NO [14]. Overloaded NO in lung tissue is considered to be one of the pathogenesis of ALI. Yehuda's [14] study suggested that glabridin could decrease LPS-induced iNOS mRNA expression by 48%. The results showed that the content of NO in LPS group was significantly higher than that in control group.

The pulmonary surfactant is a complex of lipoproteins secreted by type II alveolar epithelium. SPA has the activity of promoting alveolar macrophages and inhibiting the exudation of alveolar proteins to the pulmonary surfactant [15]. Therefore, lowered activity or the lack of SPA in lung tissue is one of the main pathogenesis in sepsis induced acute lung injury.

In infectious lung injury, the secretion of SP decreased due to the degeneration and destruction of type II alveolar epithelium. Increasing permeability of alveolar capillary results in two-way leakage of the intravascular macromolecules and SP [16]. Polymorphonuclear leukocytes and other intravascular inflammatory cells accumulate and migrate. Elastase released by these cells and plasma protein leaked out can disintegrate and denaturalize SP, which eventually attenuate surface tension relieving. In addition, the increase of pulmonary capillary permeability allows SP entering the bloodstream. SP-A also has the function of regulating macrophages, promoting chemotactic activity, enhancing phagocytosis and stimulating the production of oxygen free radicals. Thus, an increase of the oxygen free radicals in blood exacerbate the sepsis, forming a vicious circle. In this study, the levels of SPA in 12 h and 24 h LPS groups were significantly higher than that in control group, while the levels in 6 h group not. The content of SPA in LPS+GLA groups was lower than that in LPS alone groups. Taking the W/D ratio of lung into consideration, glabridin may have the potential to inhibit the degradation of pulmonary surfactant, increase the activity of SPA, decrease the permeability of pulmonary capillary, reduce the aggregation inflammatory factors and alleviate lung edema. Therefore, glabridin plays a protective role in sepsis induced lung injury in rats.

## MATERIALS AND METHODS

### Reagents

LPS, anti-phosphorylated P38MAPK antibody, anti-P38MAPK antibody, anti-phospho-ERK antibody, anti-ERK antibody were purchased from Sigma-Aldrich Corporation (Shanghai, China). Rats TNF-a, IL-18 and surfactant protein A (SPA) were purchased from Wuhan Beauty Bio Technology Co. Ltd (Wuhan, China). Malondialdehyde (MDA), superoxide dismutase (SOD), and nitric oxide (NO) kits were purchased from Nanjing Jiancheng Biological Engineering Research Institute (Nanjing, China). Light licorice was ordered from Nantong Feiyu Biological Technology Co. Ltd (Nantong, China).

### Animals

64 healthy male Wistar rats, weighing approximately 160 to 200 g, were obtained from the Laboratory Animal Center of Inner Mongolia University (Hohhot, China), SCXK (Mongolian) 2002-0001. All the rats were provided with water and chow ad libitum under standardized laboratory conditions for 1 week to adapt the environment before experiments. All the experimental protocols were approved by Animal Ethics Committee of the Affiliated Hospital of Innner Mongolia Medical University.

### Experimental protocol for acute lung injury model

All rats were randomly divided into 8 groups and each group contained 8 rats: a control group (saline), a GLA group, 3 LPS groups (6 h, 12 h, 24 h), 3 LPS+GLA groups (6 h, 12 h, 24 h). LPS (10 mg/kg) was administered intraperitoneal to induce acute lung injury, and the control group was injected with the same dose of saline. The GLA group received glabridin 30 mg/kg at the same time. Glabridin was given intragastric infusion administration 1h after intraperitoneal injection. The doses of these drugs were on the basis of previous studies and our preliminary experiments [17]. All the rats in each group were euthanized at the same time. The bloods and lung tissue specimens were collected at the prescribed time.

### Lung wet/dry weight ratio

The wet/dry (W/D) ratio was assayed in the upper lobe of right lung to assess lung edema. The upper lobes of the right lung were excised and the wet weights were determined. Then, the lungs were placed in sterile non-enzyme tube. After incubated at 80°C for 72 hours to remove all moisture, the dry weights were measured and the W/D ratio were calculated.

### Measurement of factors

The plasma and homogenate of the upper lobes of the left lungs was preserved in liquid nitrogen for the determination of different factors. The concentrations of TNF-α, IL-18, and SPA in plasma were detected using ELISA kit according to the protocol recommended by the manufacturer. MDA, SOD and NO levels in lung homegenate were respectively determined with methods of TBA, xanthine oxidase and nitric reductase.

### Histological evaluation

The right lower lungs were collected and flushed by frozen saline, and fixed with 4% formaldehyde for 24 hours at 25°C. Then, the lung tissue blocks were routinely repaired, dehydrated and embedded. After stained with hematoxylin and eosin (H&E), pathological changes of the lung tissue were observed under a light microscope.

### Immunohistochemical analysis of lung tissue p-p38MAPK and pERK expression

The lung tissue paraffin specimens underwent conventional dewaxing hydration and antigen retrieval for 12 minutes. 3% hydrogen peroxide was used to block endogenous peroxidase. 1:100 rabbit anti-rat p-p38MAPK and ERK protein kinase monoclonal antibody were added as the primary antibody for each group, then biotin-labeled secondary antibody. The negative control group was added with PBS instead of the primary antibody. The brown-yellow granules in the lung tissue were observed under a light microscope.

### Western blot analysis

The lower lobes of the left lungs were preserved in liquid nitrogen until homogenization. Proteins content was measured using Coomassie brilliant blue staining. 80 g protein samples extracted from the lungs of each group were subjected to sodium dodecyl sulfate polyacrylamide gel electrophoresis (SDS-PAGE) in a 12% gel and transferred onto nitrocellulose membrane. The nitric acid film was sealed in 2% skimmed milk powder at 25°C. After washed in TPBS and labeled with horseradish peroxidase (HRP) and DAB chromogenic reagent kit, the density of the target bands on the membrane were scanned and analyzed by a gel imaging system (Aphla, American).

### Statistical analysis

All values were expressed as mean values ± standard deviation(SD). Differences between the groups were analyzed by one-way analysis of variance (ANOVA), and paired specimen by *T*-test in SPSS 17.0 (SPSS Inc., USA). *P* values < 0.05 were considered to be statistically significant.

## CONCLUSIONS

Glabridin can attenuate acute lung injury in LPS-induced ARDS rats. Glabridin can inhibit inflammatory responses and play a protective role by inhibiting activation of the p38MAPK and ERK signal transduction pathway. Besides reducing capillary leakage, glabridin can lower SPA content and activity in lung plasma but raise the content and activity in lung tissue. Glabridin can reduce the damage of lung tissue through anti-oxidation and oxygen free radicals scavenging.
